# Bile acid signaling at the gut–vascular interface: a novel modulator of hantavirus endothelial barrier dysfunction

**DOI:** 10.3389/fcimb.2026.1883162

**Published:** 2026-07-22

**Authors:** Lei Liu, Jiyong Lin, Kai Sang, Jianping Lai, Na Huang, Peiling Zhong, Yuxiang Liu, Sheng Chen

**Affiliations:** 1 Department of Infectious Diseases, Shenzhen Traditional Chinese Medicine Hospital, The Fourth Clinical Medical College of Guangzhou University of Chinese Medicine, Shenzhen, China; 2 Department of Emergency, Shenzhen Traditional Chinese Medicine Hospital, The Fourth Clinical Medical College of Guangzhou University of Chinese Medicine, Shenzhen, China; 3 Department of Respiratory and Critical Care Medicine, Shenzhen Traditional Chinese Medicine Hospital, The Fourth Clinical Medical College of Guangzhou University of Chinese Medicine, Shenzhen, China

**Keywords:** bile acids, FXR/TGR5, gut microbiota, hantavirus, hemorrhagic fever with renal syndrome

## Abstract

Hantavirus infection triggers life-threatening hemorrhagic fever with renal syndrome (HFRS) and hantavirus cardiopulmonary syndrome (HCPS), driven by severe endothelial barrier breakdown and systemic capillary leakage. Clinical severity varies widely with undefined host regulators, and no targeted endothelial-protective treatments exist. Recent data link hantaviruses to gut microbiome remodeling, while bile acid (BA) receptors FXR and TGR5 potently inhibit NF-κB-mediated endothelial inflammation. We synthesize four core lines of evidence. First, metagenomic reports confirm hantavirus reshapes gut/lung microbiota in rodent reservoirs. Second, we re-analyzed three public GEO datasets via standardized RNA-seq/microarray pipelines: (i) GSE245916: SEOV-infected human/rat lung ECs show conserved VCAM1/ICAM1 upregulation (human VCAM1 log_2_FC=+1.17, P = 0.023; rat Icam1 log_2_FC=+0.32, padj=0.016) with unaltered FXR; (ii) GSE7271: SEOV-infected rat lung displays sustained Nfkb1 suppression (all timepoints, P<0.05) and day-15 Slc10a2 downregulation (P = 0.028); (iii) GSE270172: PUUV 3D vessel chips feature robust IL6 elevation (log_2_FC=+1.22, P = 3.1×10^-8^) and disrupted BA transporters (ABCC3 log_2_FC=−1.44, P = 7.4×10^-^¹²). TGR5 (GPBAR1) was undetectable in endothelial cells across all datasets. Third, FXR/TGR5 agonists repress NF-κB inflammation and mitigate lung vascular injury. Fourth, HTNV upregulates CH25H to block HMGCR-dependent cholesterol synthesis, depleting BA precursor substrates. We propose a unified pathogenic model: hantavirus-triggered gut dysbiosis plus virus-impaired cholesterol metabolism deplete circulating FXR/TGR5 agonistic BAs, relieving constitutive inhibition of endothelial NF-κB and monocyte NLRP3 inflammasomes to exacerbate capillary leakage. We define tiered testable predictions covering clinical multi-omics cohorts, *in vitro* receptor modulation assays and *in vivo* pharmacological interventions. This gut microbiota–BA–FXR/TGR5 axis represents a repurposable therapeutic target for hantavirus diseases, though direct causal evidence connecting BA signaling to viral vascular damage remains absent; our framework offers a rigorous testable roadmap for subsequent validation.

## Introduction

Hantaviruses (order Bunyavirales, family Hantaviridae) are globally distributed rodent-borne pathogens that cause two severe human syndromes: hemorrhagic fever with renal syndrome (HFRS) in Eurasia, caused primarily by Hantaan virus (HTNV), Seoul virus (SEOV), Puumala virus (PUUV), and Dobrava-Belgrade virus (DOBV); and hantavirus cardiopulmonary syndrome (HCPS) in the Americas, caused by Andes virus (ANDV) and *Sin Nombre* virus (SNV) (Casel et al., 2026). Hantaviruses possess a tri-segmented, negative-sense, single-stranded RNA genome: the small (S) segment encodes the nucleocapsid (N) protein; the medium (M) segment encodes the envelope glycoprotein precursor (GPC), which is cleaved into Gn and Gc; and the large (L) segment encodes the viral RNA-dependent RNA polymerase (RdRp). The N protein interacts with host NF-κB signaling, while Gn/Gc mediate receptor binding and entry via β3 integrins on endothelial cells (Casel et al., 2026).Globally, approximately 150, 000–200, 000 HFRS cases occur annually, with China bearing the highest disease burden (Li et al., 2025; Casel et al., 2026; Duan et al., 2026). Despite advances in understanding hantavirus pathogenesis, clinical management remains limited to supportive care, and no specific antiviral or host-directed therapy has been approved (Casel et al., 2026).

The pathological hallmark of hantavirus disease is not direct viral cytolysis, but rather a profound functional disruption of the microvascular endothelial barrier. Hantaviruses productively infect human microvascular endothelial cells without causing cytopathic effect or cell detachment; instead, they induce a dysregulated host inflammatory response that drives vascular leakage, the clinical manifestation of which is the triad of thrombocytopenia, capillary leak syndrome, and acute kidney injury (AKI) that defines severe HFRS (Gorbunova and Mackow, 2021; Casel et al., 2026). Monocyte-derived TNF-α, IL-1β, and IL-6 are secreted upon hantavirus sensing and act in a paracrine manner to increase endothelial permeability, an effect that is reversed by anti-TNF-α neutralizing antibody (Wang et al., 2026). IL-6 trans-signaling directly disrupts VE-cadherin organization and correlates with disease severity in HFRS patients [Maleki et al., 2025]. Soluble thrombomodulin (sTM) has been validated as a biomarker that tracks disease progression and predicts mortality in HFRS (Wei et al., 2026). At the transcriptional level, NF-κB-driven cytokine storm is recognized as the shared core pathology across the Bunyaviricetes class (Casel et al., 2026).

A fundamental and clinically consequential question remains unresolved: why do some patients infected with the same hantavirus strain develop fulminant, life-threatening capillary leak while others experience mild or subclinical illness? Host genetic factors, including HLA haplotypes and CCR5 polymorphisms, explain only a fraction of the observed variability (Kletenkov et al., 2019; Haapaniemi et al., 2025). Viral factors such as inoculum dose and strain also contribute, but do not fully account for the severity spectrum. Notably, the literature lacks a comprehensive framework integrating microbial, metabolic, and immunological determinants of hantavirus disease severity. Addressing this gap, we propose a novel hypothesis linking the gut microbiota–bile acid axis, mediated by the Farnesoid X receptor (FXR, *NR1H4*) and Takeda G protein-coupled receptor 5 (TGR5, *GPBAR1*), to endothelial barrier dysfunction. Importantly, no clinical metagenomic or metabolomic data from HFRS patients are currently available to test this hypothesis; the present article provides the conceptual framework and specific experimental predictions required to generate such data.

The gut–lung axis—the bidirectional immunological and metabolic crosstalk between the gastrointestinal and respiratory tracts mediated by the gut microbiome and its metabolites—has emerged as a critical determinant of severity in multiple viral respiratory diseases, including influenza, SARS-CoV-2, and respiratory syncytial virus (Budden et al., 2017; Özçam and Lynch, 2024). This axis operates through multiple interconnected mechanisms: (1) microbial metabolites, particularly short-chain fatty acids (SCFAs) and secondary bile acids (BAs), enter the portal and systemic circulations to modulate pulmonary immune tone through G-protein-coupled receptors (e.g., GPR41/43 for SCFAs, TGR5 for BAs) and nuclear receptors (e.g., FXR) (Zhang et al., 2012; Özçam and Lynch, 2024); (2) gut-derived cytokines and immune cells traffic through the lymphatic and circulatory systems to influence lung mucosal immunity; (3) dietary and microbial metabolites such as tryptophan derivatives activate aryl hydrocarbon receptor (AhR) signaling in lung epithelial and immune cells (Özçam and Lynch, 2024). Importantly, gut microbiota dysbiosis—characterized by reduced microbial diversity and altered BA-metabolizing capacity—can shift the systemic BA pool composition, decreasing FXR/TGR5 agonistic profiles and consequently amplifying pulmonary inflammatory responses (Zhang et al., 2012; Ridlon et al., 2016). Bile acids, in particular, represent a direct metabolic link between gut microbial ecology and remote vascular barrier function, as they are exclusively produced in the liver, modified by the gut microbiota, and delivered via the circulation to peripheral tissues including the lung and kidney capillaries (Özçam and Lynch, 2024). Despite the extensive characterization of the gut–lung axis in other viral infections, this axis remains entirely unexplored in the context of hantavirus disease. A PubMed search for (“hantavirus” OR “HFRS”) AND (“bile acid” OR “FXR” OR “TGR5” OR “*NR1H4*” OR “*GPBAR1*”) returns zero results (as of May 2026). Notably, serum BA profiles have emerged as biomarkers of disease severity and gut barrier dysfunction in other viral infections: elevated total BA and altered BA composition predict poor outcomes in COVID-19, and BA dysregulation has been linked to intestinal permeability and systemic inflammation in influenza and hepatitis (Özçam and Lynch, 2024).Complementarily, severe pulmonary inflammatory insults—including viral-induced endothelial injury—can circulate systemic pro-inflammatory signals back to the gastrointestinal tract, disrupting intestinal barrier integrity and reshaping bile acid-metabolizing commensal populations, thereby forming a self-amplifying inflammatory feedback loop. This uncharted intersection between bile acid gut–lung signaling and hantavirus-triggered pulmonary endothelial dysfunction constitutes the core mechanistic basis of our gut–vascular interface hypothesis proposed herein.

We use the term “gut–vascular interface” (GVI) to denote the systemic signaling axis through which microbiota-derived bile acid metabolites reach and modulate the function of distal microvascular endothelial beds—including those of the kidney and, in severe cases, the lung—the principal target organs in hantavirus disease. This concept is built upon the established gut–vascular barrier (GVB), first identified in the small intestine by Spadoni et al (Spadoni et al., 2015). The GVB comprises intestinal endothelial cells interconnected by tight junctions (occludin, ZO-1, cingulin, JAM-A) and adherens junctions (VE-cadherin, β-catenin), stabilized by enteric glial cells and pericytes, and restricts the passage of solutes and macromolecules above approximately 70 kDa into the bloodstream (Spadoni et al., 2015). The integrity of this barrier is maintained by the canonical Wnt/β-catenin signaling pathway, and its disruption—marked by upregulation of the endothelial glycoprotein plasmalemma vesicle-associated protein-1 (PV-1)—has been shown to facilitate bacterial translocation from the gut to distant organs, including the liver (Bertocchi et al., 2021). GVB disruption further triggers systemic innate immune activation: translocated microbial fragments and aberrant circulating BA profiles jointly stimulate tissue-resident macrophages at pulmonary and renal microvascular beds, driving local NF-κB-dependent pro-inflammatory cytokine release and amplifying endothelial barrier leakage—the core pathological phenotype of hantavirus-induced HFRS. Beyond its barrier function, the GVI serves as an endocrine-like conduit for bile acids (BAs), which are synthesized in the liver, conjugated with taurine or glycine, secreted into the duodenum, deconjugated and modified by the gut microbiota, and then reabsorbed via the portal circulation to reach systemic endothelial beds. The composition of the recirculating BA pool is therefore directly shaped by gut microbial ecology, linking intestinal dysbiosis to remote vascular sites. This concept is mechanistically distinct from the traditional “gut–lung axis” mucosal immunity framework, though the two are complementary. While the gut–lung axis emphasizes local immune cell trafficking, IgA-mediated mucosal defense, and direct microbial metabolite effects on respiratory epithelium, the GVI focuses on the endocrine-like delivery of BA receptor ligands via the portal and systemic circulation to endothelial and immune cells at remote vascular sites. We employ this terminology throughout the manuscript to distinguish the systemic BA-farnesoid X receptor (FXR, *NR1H4*)/G protein-coupled bile acid receptor 1 (TGR5, *GPBAR1*) signaling paradigm from mucosal immune cross-talk.The anatomical and functional components of the GVI are schematically illustrated in **Supplementary Figure 1**.

Bile acids signal through two major receptor systems with established roles in vascular inflammation and endothelial barrier maintenance. Farnesoid X receptor (FXR, *NR1H4*), a nuclear receptor expressed at low levels in endothelial cells, functionally antagonizes NF-κB p65 transcriptional activity through co-repressor recruitment (Zhang et al., 2012), suppressing expression of *VCAM1*, *ICAM1*, *TNF*, and *IL6*. Takeda G protein-coupled receptor 5 (TGR5, *GPBAR1*), expressed predominantly in monocytes and macrophages but below detection in quiescent endothelial cells (Perino and Schoonjans, 2015), signals through cAMP/PKA to inhibit *NLRP3* inflammasome activation and suppress TNF-α, IL-6, and IL-1β secretion (Hu et al., 2021). Both receptors are activated by bile acids: CDCA is the most potent endogenous FXR agonist, while secondary bile acids LCA and DCA are TGR5 agonists. The composition of the BA pool is directly determined by gut microbial metabolism, with bile salt hydrolase (BSH)-active bacteria serving as gatekeepers of BA receptor signaling (Ridlon et al., 2016). Elevated intestinal BA concentrations secondary to dysbiosis may directly impair barrier integrity and exacerbate systemic inflammation (Ridlon et al., 2016), reinforcing the gut–vascular axis concept.The signaling cascades of both receptors downstream of bile acid activation are schematically summarized in **Supplementary Figure 2**.

The objective of this hypothesis article is to integrate dispersed published evidence across metagenomics, transcriptomics, BA receptor pharmacology, and cholesterol metabolism to construct a testable framework linking bile acid signaling to hantavirus endothelial pathology—a connection that has not been previously proposed. Here, we synthesize four converging evidence chains to propose that the gut microbiota–bile acid–FXR/TGR5 axis functions as a host-intrinsic modulator of endothelial barrier integrity in hantavirus disease. We propose that hantavirus-induced microbiome perturbation, combined with virus-mediated disruption of cholesterol metabolism, shifts the bile acid pool away from FXR/TGR5-agonistic profiles, reducing tonic suppression of endothelial NF-κB signaling and thereby potentially amplifying vascular leakage. We outline specific, testable predictions. We emphasize that direct evidence linking BA signaling to hantavirus pathogenesis currently does not exist; this article provides a testable framework for investigation.

## Evidence supporting the gut microbiota–bile acid–FXR/TGR5 axis hypothesis

Until recently, the effect of hantavirus infection on host-associated microbial communities was entirely unknown. Two landmark studies have now established that hantaviruses may directly alter the composition of both gut and lung microbiomes in their natural rodent reservoirs, providing the foundational evidence for the first link in our hypothesis.

The first direct evidence emerged from a study of wild bank voles (*Myodes glareolus*), the natural reservoir host of PUUV. Using 16S rRNA gene sequencing of intestinal samples, the authors demonstrated that PUUV infection significantly altered both α-diversity (increased) and β-diversity of the gut microbiota, with pathogen-specific community patterns that distinguished PUUV-monoinfected animals from those co-infected with other rodent pathogens. These alterations were not attributable to host age, sex, or body condition, suggesting a virus-specific effect on gut microbial community structure (Brila et al., 2023).

Direct experimental evidence that hantavirus can infect and disrupt the gastrointestinal epithelium was provided by Witkowski et al. Using Caco-2 human intestinal epithelial monolayers, the authors demonstrated that PUUV productively infects enterocytes and causes ZO-1 tight junction protein redistribution, leading to a significant loss of epithelial barrier function as measured by transepithelial electrical resistance. This finding establishes the gastrointestinal tract as a portal of hantavirus entry and a direct target of barrier-disrupting viral pathology, providing mechanistic support for gut-derived systemic inflammatory amplification in HFRS (Witkowski et al., 2017).

This finding was recently extended by a metagenomic study of lung tissue from hantavirus-infected rodents collected at surveillance sites in Hunan Province, China (Xiong et al., 2026). The study compared the lung microbiomes of HTNV-infected *A. agrarius* and SEOV-infected *R. norvegicus* with their uninfected counterparts, revealing striking host–virus pairing specificity. HTNV infection significantly reduced lung microbial richness—as measured by observed species, ACE index, and Chao1 index—while increasing community evenness (Shannon and Simpson indices). In contrast, SEOV infection of *R. norvegicus* produced no significant change in α-diversity. β-diversity analysis showed clear separation of microbial community structure by both host rodent species and virus infection status, with the SEOV-infected rat lung microbiome bearing greater similarity to uninfected rat lungs than HTNV-infected mouse lungs did to uninfected mouse lungs (Xiong et al., 2026). Linear discriminant analysis effect size (LEfSe) identified specific taxa associated with infection status, including *Streptococcus agalactiae* enrichment in SEOV-infected rats and relative depletion of *Chlamydia* in uninfected *A. agrarius*. These host-virus species-specific patterns suggest that hantavirus-induced microbiome perturbation is not a generic inflammatory response, but a biologically specific interaction.

The gastrointestinal tract is clinically affected in acute HFRS. Nausea, vomiting, diarrhea, and abdominal pain are common presenting symptoms, and hantaviruses are classified among non-typical viral infections capable of producing gastrointestinal symptoms and epithelial barrier involvement (Crnčević et al., 2022). The gut may serve as both a target of viral pathology and a contributor to systemic inflammation in HFRS. Gut dysbiosis may impair intestinal barrier integrity, potentially enabling microbial translocation that amplifies systemic inflammation and endothelial injury.

A critical knowledge gap must be acknowledged: no study has yet profiled the gut or lung microbiome in human HFRS patients. The existing evidence derives exclusively from rodent reservoir hosts, in which infection is typically asymptomatic and persistent-an immunological state that fundamentally differs from the acute, inflammatory disease in humans (Klimaj et al., 2024; Noack et al., 2024a; Irving et al., 2026). Reservoir rodents maintain a regulatory immune profile that suppresses antiviral T cell responses through three complementary mechanisms (Noack et al., 2024a). First, upregulation of programmed death-ligand 1 (PD-L1) engages the PD-1 inhibitory receptor on cytotoxic CD8^+^ T cells, delivering an exhaustion signal that attenuates T cell effector function. Second, downregulation of beta-2 microglobulin (B2M), an essential subunit of MHC class I molecules, impairs antigen presentation to CD8^+^ T cells and reduces recognition of infected cells. Third, shifts in the JAK-STAT signaling network alter interferon-γ responsiveness, further blunting the antiviral transcriptional program. These host-adapted immune evasion strategies contrast sharply with the pro-inflammatory signaling sustained by human endothelial cells upon hantavirus infection.The downstream metabolic consequences of microbiome perturbation may therefore differ between reservoir and incidental hosts. Whether the magnitude of dysbiosis observed in rodent reservoirs is comparable to, greater than, or qualitatively distinct from that in human HFRS patients remains entirely unknown. Nevertheless, the demonstration that hantaviruses can restructure host microbial communities-even in their natural, co-evolved reservoirs-establishes biological plausibility and motivates investigation in human disease.

FXR activation protects against inflammatory lung injury and endothelial barrier failure through NF-κB suppression. FXR agonism by CDCA attenuates LPS-induced acute lung injury in mice, reducing pulmonary edema, neutrophil infiltration, and bronchoalveolar lavage fluid TNF-α, IL-6, and IL-1β levels (Zhang et al., 2012). The semi-synthetic FXR agonist obeticholic acid (OCA, approximately 100-fold more potent than CDCA) similarly alleviates LPS-induced acute lung injury through NF-κB pathway inhibition (Fei et al., 2019). FXR activation also protects pulmonary tissue from IL-6-induced ferroptosis-a regulated cell death pathway implicated in viral lung injury (Yang et al., 2024). In macrophages, TGR5 activation by secondary BAs or synthetic agonists elevates intracellular cAMP, leading to PKA-mediated suppression of NF-κB and inhibition of *NLRP3* inflammasome activation (Pols, 2014). Ginsenoside Rh1 treatment remodels the gut microbiota, reduces T-β-MCA levels (relieving FXR antagonism), and increases LCA levels (enhancing TGR5 agonism), providing direct causal evidence for the microbiota-to-BA-to-receptor signaling chain (Gao et al., 2026). Beyond endothelial and immune cells, TGR5 is abundantly expressed in enteroendocrine L cells of the distal ileum and colon, where its activation by secondary BAs stimulates GLP-1 secretion via cAMP/PKA signaling (Perino and Schoonjans, 2015). This EEC–TGR5–GLP-1 axis represents an additional gut-initiated pathway through which microbiota-derived BA metabolites could exert systemic metabolic and anti-inflammatory effects, complementing the direct endothelial and immune cell mechanisms discussed above and reinforcing the gut–vascular interface concept.

The cholesterol metabolic pathway provides a biochemical bridge between hantavirus infection and BA homeostasis. HTNV infection induces cholesterol 25-hydroxylase (*CH25H*), an interferon-stimulated gene that converts cholesterol to 25-hydroxycholesterol (25HC). 25HC inhibits HTNV by downregulating *HMGCR*, the rate-limiting enzyme of cholesterol biosynthesis (Dang et al., 2024). Since cholesterol is the obligate precursor for all BA synthesis via *CYP7A1*, *HMGCR* suppression would reduce the cholesterol substrate pool available for BA synthesis. HTNV infection of BEAS-2B lung epithelial cells alters steroid hormone and terpenoid metabolism (Ding et al., 2024), further supporting that hantavirus infection broadly perturbs host lipid metabolism. The downstream consequence for vascular permeability is indirect but mechanistically coherent: reduced BA synthesis and/or altered BA pool composition could diminish the availability of FXR and TGR5 agonists in the systemic circulation. Because both receptors tonically suppress endothelial NF-κB activity—FXR via co-repressor recruitment and TGR5 via cAMP/PKA/NLRP3 inhibition—a BA-depleted or compositionally shifted pool would weaken this tonic anti-inflammatory restraint, leaving endothelial cells more susceptible to NF-κB-driven pro-adhesive and pro-permeability signaling. This cholesterol–BA–FXR/TGR5 axis is schematically illustrated in **Supplementary Figure 3**.

In human hantavirus disease, the molecular basis of NF-κB-driven cytokine amplification differs fundamentally from reservoir host immune tolerance. Hantavirus infection of human endothelial cells activates NF-κB through multiple non-mutually exclusive pathways: viral nucleocapsid protein (NP) directly interacts with the NF-κB p65 subunit and promotes its nuclear translocation (Gorbunova and Mackow, 2021); viral glycoproteins Gn and Gc trigger Toll-like receptor 4 (TLR4)-mediated signaling upstream of IκB kinase (IKK), leading to IκBα phosphorylation and degradation (Gorbunova and Mackow, 2021; Perez et al., 2021); and virus-induced reactive oxygen species (ROS) further potentiate NF-κB transcriptional activity (Perez et al., 2021). Once activated, endothelial NF-κB drives the expression of monocyte chemoattractant protein-1 (MCP-1/CCL2), IL-6, and IL-8, recruiting and activating monocytes that amplify the inflammatory cascade through TNF-α and IL-1β secretion (Maleki et al., 2025; Wang et al., 2026). The resulting positive-feedback loop—monocyte-derived cytokines further activate endothelial NF-κB—sustains the cytokine storm that underlies capillary leakage and organ dysfunction in severe HFRS. This contrast between reservoir host NF-κB suppression and human NF-κB amplification highlights the critical role of host-specific inflammatory regulation in determining disease outcome, and provides the rationale for investigating whether bile acid receptor signaling—which tonically suppresses NF-κB in endothelial cells—is differentially modulated across host species.

Multiple converging mechanisms drive endothelial barrier disruption in hantavirus disease. A recently clarified central mechanism involves monocyte-mediated, TNF-α-dependent paracrine signaling. In a Transwell co-culture model, HTNV infection of PBMCs led to secretion of TNF-α, IL-1β, and IL-6 into the culture medium, accompanied by progressive decline in HUVEC transepithelial electrical resistance (TEER). Depletion of monocytes significantly reduced cytokine secretion and attenuated barrier disruption, while anti-TNF-α neutralizing antibody reversed the increase in endothelial permeability (Wang et al., 2026). IL-6 trans-signaling directly disrupts VE-cadherin organization and enhances endothelial permeability in hantavirus-infected primary human endothelial cells (Maleki et al., 2025). Soluble thrombomodulin, shed from the endothelial surface upon injury, predicts HFRS mortality with an AUC of 0.93 (Wei et al., 2026). Additional mechanisms include ANDV nucleocapsid protein-mediated RhoA release from RhoGDI, driving VE-cadherin phosphorylation and internalization (Gorbunova and Mackow, 2021), VEGF secretion from infected pericytes acting in a paracrine manner on endothelial cells (Perez et al., 2021), and kallikrein-kinin system activation through factor XII-dependent bradykinin liberation (Taylor et al., 2013).

## GEO datasets re-analysis: transcriptomic evidence

To further supply indirect transcriptomic evidence for the above integrated mechanistic framework, we re-analyzed three independent public GEO transcriptomic datasets covering different hantavirus strains, host species, and *in vitro* endothelial models. The first, GSE7271, a microarray study of rat lungs infected with Seoul virus at a 3-day, 15-day, and 40-day time course (n = 24, GPL341 platform). This dataset provides a unique longitudinal window into the transcriptional response of the natural reservoir host lung. Strikingly, *Nfkb1* was downregulated at all three time points (3d ΔRMA = −258, P = 0.033; 15d ΔRMA = −209, P = 0.040; 40d ΔRMA = −266, P = 0.021; Affymetrix RMA-normalized intensity scale, not log2 fold-change), suggesting suppression of NF-κB signaling in the reservoir lung—a finding consistent with the immunotolerant phenotype of natural hosts. *Il1b* was also downregulated at 3d (ΔRMA = −197, P = 0.079) and 40d (ΔRMA = −177, P = 0.115), though not reaching formal significance at the 15d time point (ΔRMA = −126, P = 0.245). *Il6* and *Tnf* showed no significant changes at any time point. These data support the concept that reservoir hosts actively suppress rather than amplify inflammatory NF-κB signaling during hantavirus infection-a stark contrast to the NF-κB-driven cytokine storm observed in human HFRS. FXR (*Nr1h4*) was weakly and non-significantly upregulated (+5.2, NS at all time points), while ASBT (*Slc10a2*), the apical sodium-dependent bile acid transporter, was significantly downregulated at 15 days (P = 0.028), suggesting potential disruption of local BA reabsorption in the infected lung. Notably, *Gpbar1* (TGR5) probe sets were absent on the GPL341 microarray platform (designed 2002–2003, prior to the discovery of TGR5 as a bile acid receptor in 2002 and its deorphanization in 2003), precluding assessment of TGR5 expression in this dataset. These findings, while indirect, provide the first glimpse of BA-related gene expression changes in hantavirus-infected lung tissue and raise the possibility that even in reservoir hosts—where disease is absent—BA transporter expression is altered during infection.

Second, to directly characterize the endothelial transcriptional response to hantavirus infection, we re-analyzed GSE245916: an RNA−seq dataset of primary human and rat lung microvascular endothelial cells infected with Seoul virus (SEOV, MOI = 1) versus mock at 48 h post−infection (n = 24) (Noack et al., 2024a). Differentially expressed genes were identified using DESeq2, focusing on endothelial adhesion molecules, inflammatory mediators, tight junction components, and BA-related transporters/receptors.

In human endothelial cells, SEOV infection induced a pro-inflammatory transcriptional program. *VCAM1* was significantly upregulated (log2FC = +1.17, P = 0.023), *ICAM1* showed a consistent trend toward upregulation (log2FC = +0.65, P = 0.075), and *CCL2* (MCP-1) was modestly elevated (log2FC = +0.54, P = 0.284). These changes indicate endothelial activation with increased capacity for leukocyte adhesion. Notably, *NFKB1* expression was essentially unchanged (log2FC = −0.03, NS), suggesting that the net transcriptional output of NF-κB activity-rather than NF-κB subunit expression itself—was elevated. Tight junction components *(CLDN5*: log2FC = −0.06, NS; *OCLN*: log2FC = +0.21, NS) and *VEGFA* (log2FC = −0.06, NS) remained stable, consistent with the non-cytopathic nature of hantavirus endothelial infection. ABCC2 (MRP2), a canalicular bile acid efflux transporter, showed a small, non-significant increase (log2FC = +0.15, P = 0.456).

In rat endothelial cells, a similar but quantitatively distinct pattern emerged. *Icam1* was significantly upregulated (log2FC = +0.32, P = 0.0013, padj = 0.016), *Ccl2* was prominently elevated (log2FC = +1.36, P = 0.009, padj = 0.056), and *Vcam1* showed a trend toward upregulation (log2FC = +0.57, P = 0.149). These data demonstrate that both human and rat endothelial cells mount an inflammatory transcriptional response to SEOV, although the rat cells show a more pronounced CCL2 induction. Critically, FXR (*Nr1h4*) was not significantly regulated (log2FC = +0.11, P = 0.70), and *Nlrp3*, a component of the inflammasome, was marginally downregulated (log2FC = −0.17, P = 0.533). *GPBAR1/TGR5* expression was below the detection threshold in both human and rat endothelial cells, consistent with the known expression pattern of TGR5 being predominantly in immune and epithelial cells rather than endothelial cells.

The key insight from GSE245916 is that SEOV activates endothelial cells in a pro-adhesive, pro-inflammatory direction (*VCAM1/ICAM1/CCL2* upregulation). Notably, FXR (*NR1H4*) mRNA levels were not significantly altered by SEOV infection in either human or rat endothelial cells. Because FXR is a ligand-activated nuclear receptor whose transcriptional activity is governed primarily by the availability of bile acid agonists rather than by its own mRNA abundance, the absence of compensatory FXR upregulation at the transcript level does not preclude the presence of functionally sufficient FXR protein. FXR expression has been documented in pulmonary endothelial cells at the protein level (Zhang et al., 2012).Rather, the critical unresolved question is whether hantavirus infection alters the composition of the bile acid pool accessible to endothelial FXR—a question that cannot be addressed by transcriptomics alone and requires targeted BA metabolomics.The below-detection expression of TGR5 in endothelial cells implies that any TGR5-mediated protective effects in hantavirus disease would necessarily operate through immune cells rather than through direct endothelial TGR5 signaling.This immune cell-centric model is supported by several independent lines of evidence. *GPBAR1* (TGR5) is robustly expressed in CD14^+^ monocytes and macrophages, where it co-localizes with the endothelial marker PECAM1 (CD31) in human vascular tissues, and its pharmacological activation redirects aortic macrophages toward an anti-inflammatory IL-10-high phenotype while reducing plaque macrophage content (Biagioli et al., 2023). In Kupffer cells and peripheral monocytes, TGR5/cAMP/PKA signaling directly inhibits *NLRP3* inflammasome assembly, suppressing caspase-1 activation and the secretion of mature IL-1β and IL-18—cytokines that are well-established mediators of endothelial barrier disruption (Perino and Schoonjans, 2015; Hu et al., 2021). The downstream consequence for the endothelium is thus indirect but mechanistically coherent: TGR5-mediated suppression of monocyte-derived IL-1β and TNF-α reduces paracrine activation of endothelial NF-κB, preserving junctional integrity and limiting vascular leakage. This paracrine protective model aligns with the established role of monocyte-derived TNF-α as the primary driver of endothelial barrier failure in hantavirus disease (Wang et al., 2026), and positions TGR5 as a negative regulator positioned upstream of the monocyte–endothelial inflammatory axis.We acknowledge that no single study has directly demonstrated TGR5-mediated protection of endothelial barrier integrity in the context of viral hemorrhagic fever; however, the convergence of independent experimental evidence from vascular biology, inflammasome immunology, and hantavirus pathogenesis supports this mechanistic framework as a testable hypothesis.

Third, to examine whether the endothelial activation pattern observed in SEOV extends to other hantavirus types and to more physiologically relevant culture systems, we re-analyzed GSE270172, an RNA-seq study of PUUV-infected 3D vessel-on-chip cultures versus mock and TNF-α-treated positive controls (n = 18) (Noack et al., 2024b). This dataset is particularly valuable because the 3D culture system preserves endothelial–extracellular matrix interactions and flow conditions that are absent in conventional monolayer cultures.

As expected, TNF-α treatment (3D vessel-on-chip, 48 h) induced a massive inflammatory transcriptional response: *VCAM1* (log2FC = +7.36, P = 1.3e-201), *ICAM1* (log2FC = +5.25, P = 1.1e-299), *CCL2* (log2FC = +5.07, P = 7.6e-61), and *IL6* (log2FC = +1.12, P = 2.8e-7) were all profoundly upregulated. This establishes the dynamic range of the 3D endothelial system and confirms its capacity to mount a robust inflammatory response.

Strikingly, PUUV infection in the same 3D system produced a fundamentally different transcriptional signature. *IL6* was significantly upregulated (log2FC = +1.22, P = 3.1e-8), consistent with the role of IL-6 in hantavirus pathogenesis (Maleki et al., 2025). However, classical endothelial adhesion molecules—*VCAM1, ICAM1*, and *CCL2*—showed no significant changes relative to mock-infected controls. This finding is in marked contrast to the *VCAM1*/*ICAM1* upregulation observed in GSE245916 monolayer cultures, and it suggests that the 3D microenvironment may modulate the endothelial inflammatory response to hantavirus infection, or that PUUV and SEOV differ in their capacity to activate endothelial adhesion molecule expression.

Critically, several BA-related genes were significantly altered by PUUV infection. *ABCC3* (MRP3), a basolateral bile acid efflux transporter, was significantly downregulated (log2FC = −1.44, P = 7.4e-12), suggesting impaired BA export capacity. *SLC27A5* (very long-chain acyl-CoA synthetase/BA-CoA ligase, involved in BA conjugation) was significantly reduced (log2FC = −0.90, P = 0.02). These coordinated changes suggest that PUUV infection may perturb BA transporter expression in endothelial cells, raising the possibility that intracellular BA homeostasis is altered during infection. ABCC3 downregulation could reduce basolateral BA efflux, potentially leading to intracellular BA accumulation and altered FXR ligand availability; SLC27A5 downregulation may impair BA conjugation efficiency, shifting the composition of the BA pool accessible to both receptors. Although direct functional validation is lacking, these transcriptional changes provide a plausible molecular link between hantavirus infection and the BA-FXR/TGR5 signaling axis. *CDH5* (VE-cadherin) was modestly upregulated (log2FC = +0.35, P = 0.005), while *VEGFA* was modestly decreased (log2FC = −0.49, P = 1.1e-4), suggesting a complex remodeling of endothelial junctional and angiogenic programs.

The GSE270172 data also highlight the dichotomy between the pro-adhesive endothelial activation induced by TNF-α and the more selective, IL-6-dominant program induced by PUUV. PUUV does not globally recapitulate the TNF-α-induced adhesion molecule activation program—a finding that has implications for the proposed therapeutic strategy, as it suggests that hantavirus-induced endothelial dysfunction may be more amenable to BA-mediated modulation than the overwhelming activation produced by direct TNF-α stimulation.We acknowledge that the GSE270172 dataset did not include bile acid or FXR/TGR5 agonist treatment conditions, and therefore does not provide direct evidence that BA signaling modulates the observed transcriptional responses. However, the rationale for including this dataset in the present analysis is twofold. First, the finding that PUUV infection alters ABCC3 and SLC27A5 expression suggests that hantaviruses perturb the cellular machinery for BA transport and handling—an observation that constitutes a plausible mechanistic link between viral infection and BA homeostasis, even in the absence of exogenous BA treatment. Second, the selective IL-6-centric transcriptional signature of PUUV, in contrast to the broad TNF-α-driven adhesion molecule storm, implies that PUUV-mediated endothelial activation may occupy a modulatable window: whereas the overwhelming activation induced by direct TNF-α may be refractory to intervention, the more restrained PUUV program could be amenable to tonic suppression by FXR/TGR5-mediated negative regulation of NF-κB and *NLRP3*. Direct testing of whether CDCA or INT-777 treatment attenuates IL-6 secretion and BA transporter gene dysregulation in PUUV-infected 3D vessel-on-chip cultures remains a high-priority experimental prediction of this hypothesis.

The transcriptional responses to hantavirus infection observed across the three independent GEO datasets are summarized in **Table 1**.

**Table 1:** GEO datasets analyzed in this study.

Dataset	Species	Model	Virus	Groups	N	Platform
GSE245916	Human/Rat	Lung microvascular EC	SEOV	Mock, SEOV (48 h)	24	RNA-seq
GSE7271	Rat	Lung tissue	SEOV	Mock, 3 d, 15 d, 40 d	24	Microarray (GPL341)
GSE270172	Human	3D vessel-on-chip	PUUV	Mock, PUUV, TNF-α	18	RNA-seq

EC, endothelial cell; SEOV, Seoul hantavirus; PUUV, Puumala hantavirus; TNF-α, tumor necrosis factor-α; GPL341, Affymetrix Rat Genome 230 2.0 Array platform.

GSE245916 includes paired human and rat lung microvascular EC infected with SEOV for 48 h. GSE7271 is a longitudinal microarray study of rat lung tissue following SEOV infection over 40 days. GSE270172 is a 3D vessel-on-chip model of human endothelial cells infected with PUUV and treated with TNF-α. N = total number of samples across all groups (mock plus infected/time points). Key findings from the re-analyses include: (1) SEOV induces a consistent pro-adhesive endothelial transcriptional program (*VCAM1*/*ICAM1* upregulation) in both human and rat ECs (GSE245916), while the bile acid-responsive nuclear receptor FXR (*NR1H4*) shows no compensatory upregulation at the mRNA level; (2) the reservoir host (rat) actively suppresses rather than amplifies NF-κB signaling during SEOV infection, with coordinated downregulation of *Nfkb1* across all time points (GSE7271); and (3) PUUV infection in a physiologically relevant 3D vessel-on-chip model does not activate canonical TNF-α-dependent adhesion pathways, yet significantly alters the expression of bile acid transporter genes ABCC3 and SLC27A5 (GSE270172), suggesting a perturbation of BA handling machinery independent of classical inflammatory signaling.

**Figure 1:**
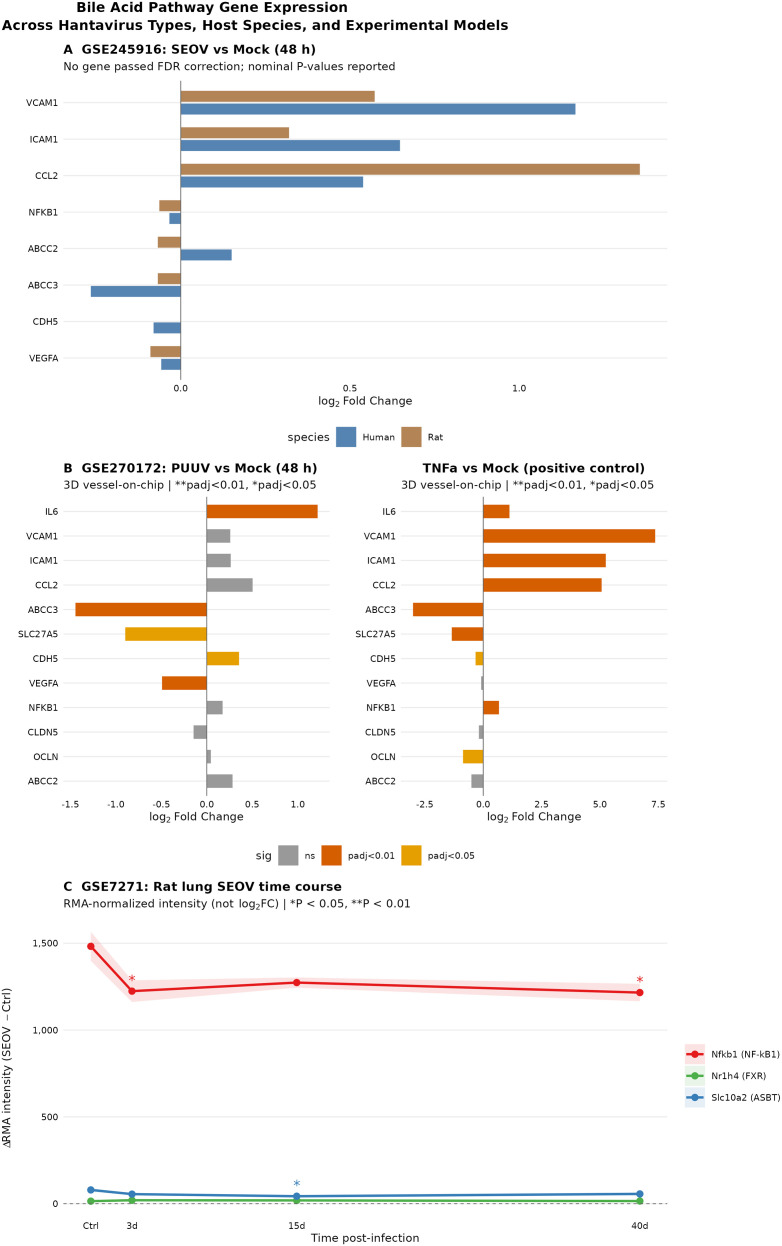
Bile acid pathway gene expression across hantavirus types, host species, and experimental models. Transcriptomic re-analyses reveal conserved endothelial inflammatory activation with concurrent BA transporter perturbation across three independent GEO datasets. **(A)** Log_2_ fold changes in human (blue) and rat (brown) lung microvascular endothelial cells infected with Seoul virus (SEOV) versus mock at 48 h (GSE245916). No gene reached the DESeq2 default FDR threshold; nominal P-values are reported. **(B)** Log_2_ fold changes in a 3D vessel-on-chip model infected with Puumala virus (PUUV, left) or treated with TNF-α (positive control for endothelial activation, right) versus mock at 48 h (GSE270172). Gray = non-significant, yellow = padj < 0.05, orange = padj < 0.01. **(C)** RMA-normalized expression (mean ± SEM) of Nfkb1 (NF-κB1), Nr1h4 (FXR), and Slc10a2 (ASBT) in rat lung tissue following SEOV infection over a 40-day time course (GSE7271). RNA-seq data were analyzed using DESeq2 with Benjamini-Hochberg correction; microarray data with limma eBayes. *raw P < 0.05, **padj < 0.01. Data re-analyzed from GSE245916 (Noack et al., 2024a), GSE270172 (Noack et al., 2024b), and GSE7271.

To synthesize the transcriptomic findings across the three GEO datasets, we constructed a comparative cross-dataset visualization (**Figure 1**). The analysis reveals three conserved patterns: (1) endothelial inflammatory activation (*VCAM1/ICAM1/CCL2/IL6* upregulation) across hantavirus types, although the magnitude and specific gene repertoire vary by model system; (2) absence of compensatory FXR*/NR1H4* upregulation in endothelial cells; and (3) consistent perturbation of BA transporter expression (*ABCC2* trend in GSE245916, *ABCC3* downregulation in GSE270172, *Slc10a2* downregulation in GSE7271), suggesting that BA handling may be altered during hantavirus infection across virus types, host species, and model systems. *GPBAR1/TGR5* expression was below detection in endothelial cells from both GSE245916 and GSE270172, consistent with its known predominant expression in monocytes and macrophages, indicating that TGR5-mediated effects in hantavirus disease would require immune-cell intermediaries. Together, the three datasets establish that (i) hantavirus infection induces conserved endothelial inflammatory programs across hantavirus species and model systems; (ii) BA transporter genes are recurrently perturbed without compensatory FXR upregulation; and (iii) TGR5 is absent from the endothelial compartment. Despite immunological differences between reservoir and human hosts, the conserved patterns of microbiome and BA pathway perturbation across species support the biological plausibility of the gut microbiota–BA–FXR/TGR5 axis as a host-modulating pathway in hantavirus disease, though whether these findings can be extrapolated to human HFRS remains to be tested in clinical cohorts.

## The hypothesis: integration and therapeutic implications

### Core hypothesis

Hantavirus infection, through combined effects on host gut microbiota composition and cholesterol metabolism, depletes the circulating bile acid pool of FXR and TGR5 agonists, thereby reducing tonic suppression of endothelial NF-κB and monocyte *NLRP3* signaling—a mechanism that may contribute to the severity of capillary leak and organ dysfunction in HFRS.

**Figure 2:**
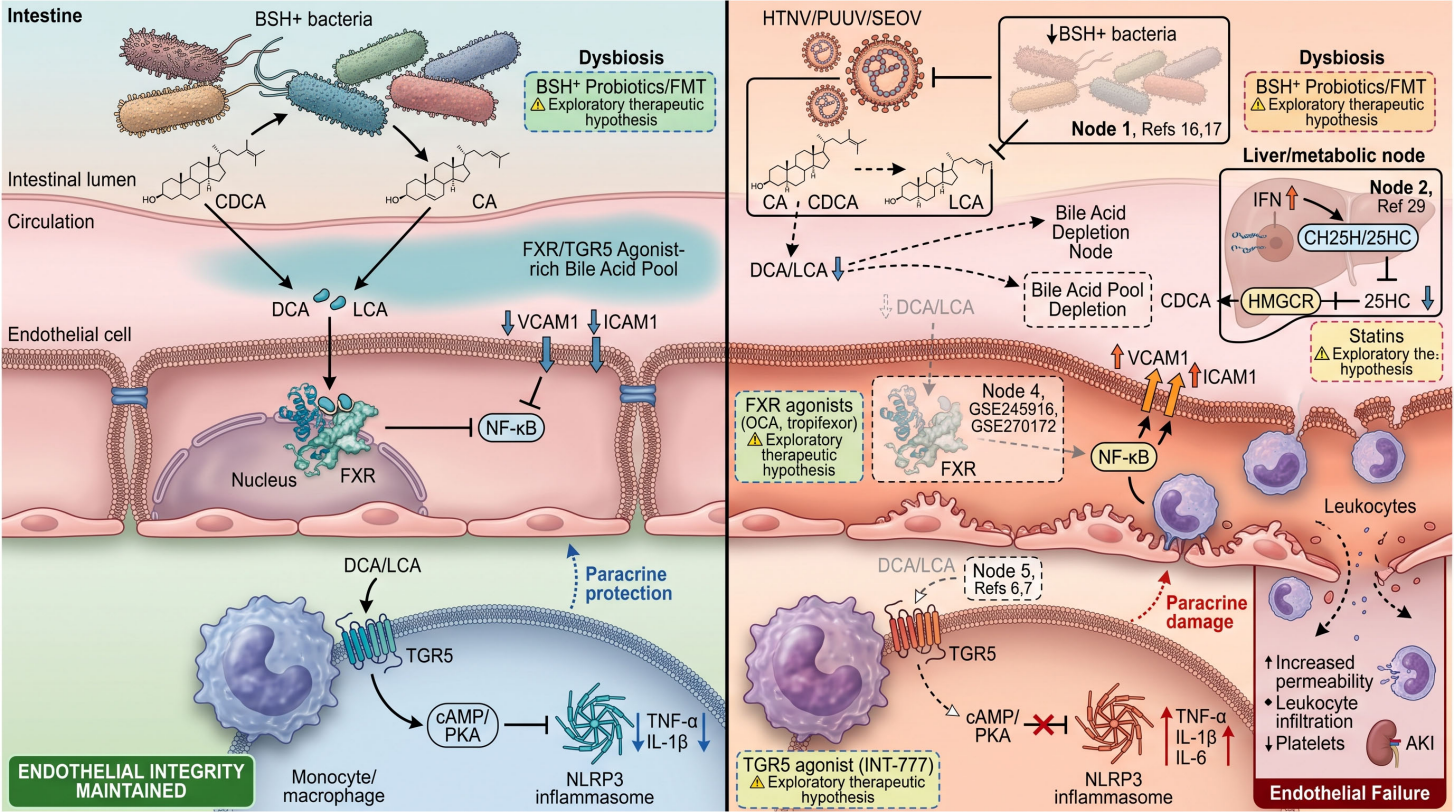
Gut microbiota–bile acid–FXR/TGR5 axis in hantavirus endothelial barrier dysfunction. (Left) Homeostasis. BSH-active gut bacteria convert primary to secondary bile acids, maintaining FXR (CDCA) and TGR5 (LCA, DCA) agonist supply. Endothelial FXR tonically suppresses NF-κB, keeping VCAM1/ICAM1 low and the vascular barrier intact. Monocyte/macrophage TGR5 activates cAMP/PKA, inhibiting NLRP3 inflammasome assembly and restraining TNF-α, IL-1β, and IL-6 secretion for paracrine endothelial protection. (Right) Hantavirus pathology. HTNV/PUUV/SEOV infection alters the gut microbiome (rodent evidence, no human validation (Witkowski et al., 2017; Brila et al., 2023)), reducing BSH-active bacteria and secondary BA production. Concurrently, virus-induced CH25H upregulation generates 25HC, suppressing HMGCR-mediated cholesterol biosynthesis and depleting the primary BA precursor pool (Dang et al., 2024). The combined effects lower circulating FXR/TGR5 agonist availability (predicted). Diminished endothelial FXR signaling relieves NF-κB suppression, upregulating VCAM1/ICAM1 (GSE245916, GSE270172), while reduced monocyte TGR5 signaling permits NLRP3 inflammasome activation and elevates monocyte-derived TNF-α, IL-1β, and IL-6, driving paracrine endothelial barrier disruption (Maleki et al., 2025; Wang et al., 2026). This paracrine axis cannot be recapitulated in pure endothelial monocultures, consistent with GSE270172. Convergent endothelial failure leads to vascular leak, thrombocytopenia, and acute kidney injury. Colored dashed boxes=exploratory therapeutic hypotheses (FXR/TGR5 agonists, probiotics/FMT, statins) without experimental verification in hantavirus models. Solid lines/boxes = experimentally validated; dashed = mechanistic extrapolations requiring direct validation. Abbreviations: AKI, acute kidney injury; BA, bile acid; BSH, bile salt hydrolase; cAMP, cyclic adenosine monophosphate; CDCA, chenodeoxycholic acid; CH25H, cholesterol 25-hydroxylase; DCA, deoxycholic acid; FMT, fecal microbiota transplantation; FXR, farnesoid X receptor; HMGCR, 3-hydroxy-3-methylglutaryl-CoA reductase; HTNV, Hantaan virus; IL, interleukin; LCA, lithocholic acid; NF-κB, nuclear factor kappa B; NLRP3, NLR family pyrin domain containing 3; OCA, obeticholic acid; PKA, protein kinase A; PUUV, Puumala virus; SEOV, Seoul virus; TGR5, Takeda G protein-coupled receptor 5; TNF-α, tumor necrosis factor alpha; 25HC, 25-hydroxycholesterol.

We propose the detailed mechanistic chain as illustrated in **Figure 2**. Briefly, hantavirus infection induces gut and lung microbiota dysbiosis to restrict secondary bile acid generation, while triggering *CH25H*-mediated cholesterol metabolism disturbance to inhibit primary bile acid synthesis. The dual alterations jointly reduce endogenous FXR and TGR5 agonists, subsequently removing the inhibitory restraint on pro-inflammatory cascades and accelerating vascular injury progression.

Together, these effects could amplify endothelial barrier failure, vascular leakage, and clinical severity in hantavirus disease. Our hypothesis does not exclude the well-documented direct cytopathic and receptor-mediated effects of hantavirus infection on endothelial cells (e.g., ANDV nucleocapsid protein–RhoA–VE-cadherin internalization (Gorbunova and Mackow, 2021); VEGF secretion from infected pericytes (Perez et al., 2021)). Rather, we propose that BA-FXR/TGR5 signaling constitutes a parallel, microbiome-dependent modulator of endothelial susceptibility to injury.

Conversely, pharmacological restoration of FXR/TGR5 signaling may attenuate hantavirus-induced endothelial barrier failure. Specifically:

- FXR agonists (OCA, GW4064, tropifexor, cilofexor): may suppress endothelial NF-κB activity, reducing *VCAM1/ICAM1* expression and thereby limiting monocyte adhesion and paracrine TNF-α/IL-6-mediated barrier disruption. The observation that FXR is expressed (albeit at low levels) in endothelial cells (GSE245916) and that FXR activation protects against LPS-induced ALI (Zhang et al., 2012; Fei et al., 2019) provides the translational rationale.
- TGR5 agonists (INT-777, LCA): may suppress monocyte/macrophage proinflammatory cytokine secretion through cAMP/PKA/NF-kB signaling (Haselow et al., 2013) and inhibit *NLRP3* inflammasome activation, thereby reducing the paracrine inflammatory stimuli that drive endothelial permeability. Given that TGR5 is below detection in endothelial cells, TGR5-based strategies would target immune cell function rather than direct endothelial protection.
- Microbiome-directed interventions (BSH-active probiotics, fecal microbiota transplantation, prebiotics): may restore BA pool composition toward FXR/TGR5-agonistic profiles, providing endogenous receptor activation that mimics the effects of pharmacological agonists.
- Combined strategies (e.g., FXR agonist + BSH-active probiotic): may provide synergistic protection by simultaneously addressing the host-side (CYP7A1 substrate deficiency) and microbiome-side (BSH/7α-dehydroxylase deficiency) contributors to BA pool dysregulation.

The evidentiary status of each component in this model is summarized in **Supplementary Table 1**, which distinguishes established findings with published experimental support from hypothetical extrapolations that await direct validation.

The central hypothesis can be decomposed into four sub-hypotheses, each generating specific, falsifiable predictions:

Sub-Hypothesis H1 (Microbiome → BA Pool): Hantavirus-induced gut and lung microbiota dysbiosis may alter the composition of the circulating and tissue BA pool, with a shift away from secondary BAs (DCA, LCA) toward primary BAs (CA, CDCA), reflecting reduced bacterial BSH and 7α-dehydroxylase activity.

Sub-Hypothesis H2 (BA Pool → FXR/TGR5 Tone): The altered BA pool may reduce FXR and TGR5 signaling in target tissues. Serum FGF19 (a direct FXR target gene product secreted by the ileum) should be lower in severe versus mild HFRS, reflecting reduced intestinal FXR activation. FXR and TGR5 target gene expression in PBMCs should be inversely correlated with disease severity.

Sub-Hypothesis H3 (Reduced FXR/TGR5 → Endothelial Injury): Reduced FXR signaling in endothelial cells may diminish suppression of NF-κB-driven *VCAM1/ICAM1* expression and cytokine production. Reduced TGR5 signaling in monocytes may diminish cAMP-mediated suppression of *NLRP3* inflammasome activation and TNF-α/IL-6 secretion. The net effect should be increased endothelial permeability, elevated sTM, and worsened clinical outcomes.We note that this hypothesis does not require hantavirus to directly induce endothelial TNF-α production. The GSE270172 data demonstrate that PUUV infection of endothelial cells in isolation does not recapitulate a TNF-α-dependent adhesion molecule program—consistent with the established model that monocyte-derived, rather than endothelial-autonomous, TNF-α is the principal paracrine driver of capillary leak in hantavirus disease^3^. The role of TGR5 signaling in our model is therefore to restrain monocyte TNF-α secretion, indirectly protecting the endothelium from paracrine inflammatory damage, rather than to modulate endothelial-autonomous TNF-α production.We further propose that this relationship operates as a bidirectional positive-feedback loop rather than a unidirectional causal chain. GVI dysfunction and hantavirus-induced cholesterol depletion shift the BA pool away from FXR/TGR5-agonistic profiles, reducing tonic suppression of monocyte *NLRP3* and NF-κB. The resulting increase in monocyte-derived TNF-α and IL-1β further disrupts endothelial barrier integrity, which in turn exacerbates GVI dysfunction and permits greater translocation of microbial products—thereby sustaining and amplifying the inflammatory cycle.

Sub-Hypothesis H4 (Cholesterol → BA Metabolic Bridge): HTNV-induced *CH25H* expression may reduce hepatic cholesterol availability for *CYP7A1*, decreasing primary BA synthesis. Serum C4 (7α-hydroxy-4-cholesten-3-one, a biomarker of BA synthesis) should be lower in acute HFRS compared to convalescent samples. Combined with microbiota-driven changes (H1), this could create a “double-hit” on the BA pool.

To guide the prioritization of future experimental validation, we present a comprehensive evidence matrix (**Table 2**) that catalogues all component claims of the hypothesis by evidence chain, documents the supporting literature with PMIDs, and classifies each finding by its evidence level relative to hantavirus disease.

**Table 2:** Evidence matrix for the gut microbiota–bile acid–FXR/TGR5 axis hypothesis in hantavirus disease.

Evidence chain	Key finding	Species/model	Ref.	Year	Study type	Evidence level
Virus → Microbiota	PUUV alters wild rodent gut microbiota (α- and β-diversity changes)	Bank vole (*Myodes glareolus*), wild	(Brila et al., 2023)	2023	16S metagenomics	Direct
	HTNV reduces lung microbial richness; SEOV shows no significant change	*A. agrarius* (HTNV), R. norvegicus (SEOV), wild	(Xiong et al., 2026)	2026	Lung metagenomics	Direct
	Hantaviruses classified as gastrointestinal dysbiosis agents	Human (review)	(Crnčević et al., 2022)	2022	Review	Contextual
Endothelial Injury	Monocyte-derived TNF-α increases EC permeability; reversed by anti-TNF-α	Human PBMC + HUVEC co-culture	(Wang et al., 2026)	2026	*In vitro* co-culture	Direct
	IL-6 trans-signaling disrupts VE-cadherin and endothelial barrier	Human primary endothelial cells	(Maleki et al., 2025)	2025	*In vitro* + patient	Direct
	Soluble thrombomodulin predicts HFRS mortality (AUC = 0.93)	Human HFRS patients	(Wei et al., 2026)	2026	Retrospective clinical	Direct
	Species-specific endothelial responses: human vs. rat EC transcriptomes	Human + rat lung microvascular EC	(Noack et al., 2024a)	2024	RNA-seq	Direct
	NF-κB dysregulation identified as core Bunyaviricetes pathology	Multiple (review)	(Casel et al., 2026)	2026	Review	Authoritative
Transcriptomic BA Evidence	Human ECs: *VCAM1*↑* (log2FC = +1.17, raw P = 0.023), *ICAM1*↑ (+0.65, P = 0.075), ABCC2 trend	Human lung microvascular EC	GSE245916	2024	RNA-seq (this study)	Direct
	Rat ECs: Icam1↑** (log2FC = +0.32, padj = 0.016), Ccl2↑ (+1.36, padj = 0.056), *Nr1h4* NS	Rat lung microvascular EC	GSE245916	2024	RNA-seq (this study)	Direct
	Rat lung: *Nfkb1*↓* (3d, 15d, 40d; RMA scale), *Slc10a2*↓* (15d, P = 0.028)	Rat (Rattus norvegicus), lung tissue	GSE7271	—	Microarray (this study)	Direct
	3D vessel-on-chip: *IL6*↑*** (log2FC = +1.22, P = 3.1e-8), ABCC3↓*** (−1.44, P = 7.4e-12), SLC27A5↓* (−0.90)	Human 3D vessel-on-chip	GSE270172	2024	RNA-seq (this study)	Direct
FXR/TGR5 Protection	FXR activation protects lung from LPS-induced acute injury via NF-κB suppression	Mouse (C57BL/6)	(Zhang et al., 2012)	2012	*In vivo*	Mechanistic
	OCA attenuates LPS-ALI through NF-κB inhibition	Mouse (C57BL/6)	(Fei et al., 2019)	2019	*In vivo*	Therapeutic proof
	FXR protects pulmonary tissue from IL-6-induced ferroptosis	Mouse	(Yang et al., 2024)	2024	*In vivo*	Mechanistic
	Ginsenoside Rh1 remodels microbiota→BA pool→FXR/TGR5 signaling: causal chain demonstrated	Mouse (T2D model)	(Gao et al., 2026)	2026	*In vivo*	Causal evidence
Metabolic Bridge	25-Hydroxycholesterol inhibits HTNV via HMGCR-mediated cholesterol suppression	BEAS-2B (human lung epithelial)	(Dang et al., 2024)	2024	*In vitro*	Direct
	HTNV alters steroid and terpenoid metabolism in BEAS-2B epithelial cells	BEAS-2B (human lung epithelial)	(Ding et al., 2024)	2024	Transcriptomics	Indirect
	Cholesterol → CYP7A1 → primary bile acids: core biochemical pathway	N/A (biochemical pathway)	—	—	Established biochemistry	Fundamental

P-value notation: *P < 0.05 (raw); **P < 0.01 or padj < 0.05; ***P < 0.001. GSE7271 values are on the Affymetrix RMA scale (not log2 fold-change).

Evidence Level is classified as: Direct (primary empirical evidence within the hantavirus system); Mechanistic (strong causal evidence in other disease models); Therapeutic proof (interventional evidence in preclinical models); Causal evidence (demonstrated complete causal chain); Contextual (supporting evidence from related systems); Authoritative (comprehensive review); Fundamental (established biochemical principles).

## Testable predictions and limitations

The hypothesis generates specific, falsifiable predictions that can be tested using existing technologies and clinically accessible samples. **Table 3** presents these predictions in order of feasibility and clinical impact, with suggested experimental systems.

**Table 3:** Prioritized, falsifiable predictions generated by the gut microbiota–bile acid–FXR/TGR5 axis hypothesis.

#	Prediction	Rationale	Test system	Expected if supported	Expected if refuted
1	HFRS patients have reduced gut microbial α-diversity and altered β-diversity	Per H1, rodent reservoir studies (Brila et al., 2023, Xiong et al., 2026) confirm hantavirus triggers gut and lung microbiome dysbiosis. Unlike asymptomatic immunotolerant rodents, human HFRS develops severe acute systemic inflammation. If microbial disturbance represents a universal viral effect, dysbiosis will be detectable and exacerbated in human patients.	16S rRNA/metagenomics on fecal samples (acute vs. convalescent vs. healthy controls)	Lower Shannon/Chao1 in severe vs. mild HFRS; β-diversity separation by severity	No difference between groups
2	Fecal and serum secondary/primary BA ratio is decreased in acute HFRS	Per H2, gut dysbiosis reduces BSH-active and 7α-dehydroxylase-expressing commensal bacteria (Ridlon et al., 2016), impairing the conversion of primary BAs (CA, CDCA) to secondary BAs (DCA, LCA). This shifts the BA pool toward primary BAs and away from TGR5-agonistic secondary BAs.	LC-MS/MS BA profiling on paired fecal and serum samples	Lower DCA/CA, LCA/CDCA ratios in severe patients, normalizing during convalescence	No difference or opposite direction
3	Serum FGF19 is lower in severe HFRS and correlates inversely with sTM	Per H2 and H3, FGF19 acts as a direct ileal FXR target and circulating biomarker of intestinal FXR activity (Zhang et al., 2012). Depletion of FXR agonistic BAs blunts ileal FXR signaling and lowers FGF19 secretion. Since FXR constitutively represses endothelial NF-κB (Zhang et al., 2012), reduced FGF19 will correlate with elevated sTM, a validated endothelial damage biomarker (Wei et al., 2026).	ELISA (FGF19, sTM) on acute-phase serum	Negative correlation between FGF19 and sTM/SOFA; FGF19 rises during recovery	No correlation
4	OCA pretreatment reduces SEOV/PUUV-induced endothelial permeability *in vitro*	Per H3, CDCA and the synthetic FXR agonist OCA repress NF-κB-mediated *VCAM1*/*ICAM1* upregulation and alleviate endothelial inflammatory injury in LPS preclinical models (Zhang et al., 2012, Fei et al., 2019). As hantavirus endothelial hyperpermeability is also driven by NF-κB hyperactivation (Casel et al., 2026), OCA pretreatment is predicted to alleviate barrier leakage.	TEER + FITC-dextran permeability assay in hantavirus-infected HLMVEC co-treated with OCA	OCA dose-dependently attenuates permeability increase and reduces *VCAM1* expression	No effect of OCA
5	OCA reduces monocyte TNF-α/IL-6 secretion in hantavirus monocyte–EC co-culture	Per H3, monocyte-derived TNF-α is the principal paracrine driver of endothelial barrier failure in hantavirus disease (Wang et al., 2026). FXR agonism suppresses endothelial NF-κB (Zhang et al., 2012); TGR5/cAMP/PKA signaling inhibits *NLRP3* inflammasome assembly and TNF-α/IL-6 secretion in monocytes (Perino and Schoonjans, 2015, Hu et al., 2021). Combined receptor activation should reduce both the paracrine inflammatory stimulus and the endothelial response, jointly preserving barrier integrity.	Transwell co-culture (PBMC + EC, HTNV-infected) with OCA treatment; multiplex cytokine assay	Significant reduction in TNF-α, IL-6, and sTM vs. vehicle control	No reduction
6	FXR agonist improves survival and reduces vascular leak in a hantavirus animal model	Per H3, OCA mitigates LPS-triggered acute lung injury (Fei et al., 2019), and FXR activation blocks IL-6-dependent pulmonary ferroptosis (Yang et al., 2024) in murine models. If the BA–FXR regulatory axis governs hantavirus-triggered systemic vascular leakage, pharmacological FXR agonism will improve survival in lethal hantavirus animal models.	Syrian hamster ANDV model or HTNV suckling mouse model + OCA (10–30 mg/kg/day)	Improved survival, reduced pulmonary edema (wet/dry ratio), lower sTM	No survival benefit
7	Serum C4 (BA synthesis marker) is reduced in acute HFRS vs. convalescence	Per H4, HTNV-induced CH25H upregulation generates 25HC, which suppresses HMGCR and depletes cellular cholesterol (Dang et al., 2024). Since cholesterol is the obligate precursor for all BA synthesis via CYP7A1, reduced cholesterol availability should lower hepatic BA synthesis. Serum C4 (7α-hydroxy-4-cholesten-3-one) is a direct, validated biomarker of CYP7A1 activity *in vivo*.	LC-MS/MS C4 measurement on paired acute/convalescent serum	Lower C4 in acute phase, rising during recovery	No change or opposite pattern
8	Hepatic *CYP7A1* expression is reduced in HTNV-infected animals	Per H4 (complementary to Prediction 7), the CH25H–25HC–HMGCR cascade depletes cholesterol, the mandatory substrate for CYP7A1-dependent primary bile acid synthesis (Dang et al., 2024). As CYP7A1 catalyzes the rate-limiting step of hepatic BA biogenesis, persistent cholesterol depletion will downregulate hepatic CYP7A1 at mRNA and protein levels.	qPCR/Western blot on liver tissue from HTNV-infected mice	Decreased *CYP7A1* mRNA and protein vs. mock-infected	No change

Several important limitations must be considered. First, the evidence for hantavirus-induced microbiome alteration derives exclusively from rodent reservoir hosts^16,18^, not from human HFRS patients; reservoir microbiota may respond differently than human microbiota—a rodent reservoir vs. human extrapolation gap that limits direct inference. Second, the proposed causal chain from dysbiosis through BA pool perturbation to altered FXR/TGR5 signaling is mechanistically plausible but unproven; microbiome changes could be an epiphenomenon of severe illness rather than a causal contributor. Third, alternative microbiome-mediated mechanisms—including SCFA-GPR41/43 signaling, TMAO production, and direct microbial translocation—could influence HFRS outcomes independently of BA signaling. Our hypothesis does not exclude these alternatives; rather, it identifies the BA–FXR/TGR5 axis as a specific, testable candidate pathway. Fourth, TGR5 expression below endothelial detection limits means that TGR5-mediated effects would require immune cell intermediaries, adding a layer of complexity to the therapeutic hypothesis. The implication is that TGR5 agonists would need to reach tissue-resident or circulating monocytes/macrophages, not directly target the endothelium. Fifth, additional signaling pathways—including MAPK cascades, apoptotic signaling, and coagulation pathway activation—may independently or synergistically drive hantavirus endothelial pathology. The relative contribution of BA-FXR/TGR5 signaling vis-à-vis these pathways requires systematic experimental dissection. Sixth, the safety of FXR/TGR5 modulators in the context of acute multi-organ dysfunction and AKI has not been established and would require careful Phase I evaluation. Finally, a critical possibility must be explicitly acknowledged: the BA pool may be unchanged in HFRS compared to convalescent or healthy controls. The hypothesis would be falsified if HFRS patient BA profiling reveals no difference from convalescent or healthy controls. A negative result would still be scientifically valuable by excluding the BA–FXR/TGR5 axis as a contributor to HFRS pathogenesis and redirecting attention to alternative microbiome-mediated mechanisms.

Taken together, this prediction panel enables a stepwise, progressively resource-intensive validation path—from clinical sample collection to *in vitro* pharmacology to *in vivo* animal models—ensuring that each phase is gated on the success of the preceding one and that null results at any stage can efficiently redirect research resources.

## Conclusion

We have proposed that the gut microbiota–bile acid–FXR/TGR5 axis functions as a host-intrinsic modulator of endothelial barrier integrity in hantavirus disease—a condition for which no specific endothelial-protective therapy currently exists. This framework integrates four previously disconnected evidence chains: (1) metagenomic evidence that hantavirus infection may alter host gut and lung microbiota (Brila et al., 2023; Xiong et al., 2026); (2) transcriptomic re-analyses of three GEO datasets (GSE245916, GSE7271, GSE270172) revealing conserved patterns of endothelial inflammatory activation (*VCAM1/ICAM1/CCL2/IL6* upregulation) with concurrent perturbation of BA transporter expression *(ABCC3, Slc10a2*) and absence of compensatory FXR upregulation; (3) robust preclinical evidence that FXR/TGR5 activation suppresses the same NF-κB-driven inflammatory cascades and protects endothelial and pulmonary barriers (Zhang et al., 2012; Fei et al., 2019; Yang et al., 2024); and (4) the cholesterol–BA metabolic pathway (Dang et al., 2024) providing a biochemical bridge between known hantavirus-induced metabolic reprogramming and BA signaling perturbation.

The hypothesis is testable, falsifiable, and—if validated—could open a new class of host-directed adjunctive therapies for a disease that currently has only supportive care. We urge the hantavirus research community, particularly clinical centers in endemic regions, to incorporate microbiome analysis and BA metabolomics into prospective HFRS cohort studies. These data, whether they support or refute our hypothesis, will fill a critical knowledge gap and may reveal whether the ancient metabolic machinery of bile acid signaling—a system evolved to integrate nutritional status with immune and barrier function—is disrupted during acute viral infection in ways that are therapeutically tractable.

## Data Availability

Publicly available datasets were analyzed in this study. This data can be found here: All datasets analyzed in this study are publicly available from the NCBI Gene Expression Omnibus (GEO): GSE245916 (https://www.ncbi.nlm.nih.gov/geo/query/acc.cgi?acc=GSE245916), GSE7271 (https://www.ncbi.nlm.nih.gov/geo/query/acc.cgi?acc=GSE7271), and GSE270172 (https://www.ncbi.nlm.nih.gov/geo/query/acc.cgi?acc=GSE270172). All re-analyzed summary statistics and code are available from the corresponding authors upon reasonable request.

## Supplementary material

Supplementary Table 1 Established versus hypothetical components of the proposed gut microbiota–bile acid–FXR/TGR5 axis hypothesis in hantavirus disease.

Supplementary Figure 1 The gut–vascular interface (GVI) as a systemic signaling axis linking gut microbiota to remote endothelial beds. **(A)** The gut–vascular barrier (GVB), first described by Spadoni et al., comprises intestinal endothelial cells connected by tight junctions (occludin, ZO-1, cingulin, JAM-A) and adherens junctions (VE-cadherin, β-catenin), supported by enteric glial cells and pericytes, and restricts macromolecular passage above ~70 kDa. Wnt/β-catenin signaling maintains barrier integrity; its disruption drives PV-1 upregulation, facilitating bacterial translocation to the liver, spleen, and distant organs. Translocated microbial fragments and aberrant circulating bile acid profiles jointly activate tissue-resident macrophages at pulmonary and renal microvascular beds, triggering local NF-κB-dependent cytokine release and amplifying endothelial leakage—the core pathology of HFRS. **(B)** Bile acids are synthesized in the liver, conjugated with taurine or glycine, secreted into the duodenum, and remodeled by gut microbiota (BSH-mediated deconjugation and 7α-dehydroxylation) before reabsorption via the portal vein. The circulating BA pool composition is therefore directly shaped by gut microbial ecology. **(C)** The GVI operates as an endocrine-like conduit: BA receptor ligands are delivered via the systemic circulation to FXR (low endothelial expression) and TGR5 (macrophage-enriched) at remote vascular sites, distinguishing this paradigm from the local mucosal immune trafficking of the traditional gut–lung axis. Abbreviations: BA, bile acid; BSH, bile salt hydrolase; FXR, farnesoid X receptor; GVB, gut–vascular barrier; GVI, gut–vascular interface; HFRS, hemorrhagic fever with renal syndrome; NF-κB, nuclear factor kappa B; PV-1, plasmalemma vesicle-associated protein-1; TGR5, Takeda G protein-coupled receptor 5.

Supplementary Figure 2 Schematic of FXR and TGR5 signaling cascades triggered by bile acids. Hepatic bile acids remodeled by gut microbiota activate two anti-inflammatory receptors: membrane TGR5 (GPBAR1) and nuclear FXR (*NR1H4*). (**A)** (TGR5): Secondary bile acids (LCA > DCA) bind macrophage-enriched TGR5, triggering Gs-cAMP-PKA signaling. Activated PKA blocks *NLRP3* inflammasome assembly and Caspase-1 maturation, reducing IL-1β/IL-18 release; PKA also represses TNF-α and IL-6 secretion. TGR5 is barely detectable in quiescent endothelial cells. **(B)** (FXR): CDCA is the dominant FXR agonist. Nuclear FXR forms an RXR heterodimer to induce SHP, BSEP and FGF19 transcription. Meanwhile, FXR recruits co-repressors to inhibit NF-κB p65, lowering endothelial *VCAM1*, *ICAM1*, TNF-α and IL-6 expression. FXR exhibits low endothelial expression. **(C)** Intestinal BSH-expressing commensals drive primary-to-secondary bile acid conversion, shaping systemic BA pools that supply ligands for both receptors. Abbreviations: BA, bile acid; BSH, bile salt hydrolase; cAMP, cyclic adenosine monophosphate; CDCA, chenodeoxycholic acid; DCA, deoxycholic acid; FXR, farnesoid X receptor; *GPBAR1*, G protein-coupled bile acid receptor 1; IL, interleukin; LCA, lithocholic acid; NF-κB, nuclear factor kappa B; *NLRP3*, NLR family pyrin domain containing 3; PKA, protein kinase A; RXR, retinoid X receptor; SHP, small heterodimer partner; BSEP, bile salt export pump; FGF19, fibroblast growth factor 19; TGR5, Takeda G protein-coupled receptor 5; TNF-α, tumor necrosis factor alpha.

Supplementary Figure 3 Cholesterol metabolic pathway linking hantavirus infection to bile acid deficiency and vascular permeability. HTNV infection upregulates cholesterol 25-hydroxylase (CH25H) via interferon signaling, driving the conversion of cholesterol to 25-hydroxycholesterol (25HC). 25HC suppresses HMGCR, the rate-limiting enzyme of cholesterol biosynthesis, thereby reducing the cholesterol substrate pool. Since cholesterol is the obligate precursor for all bile acid (BA) synthesis via CYP7A1, cholesterol depletion leads to diminished BA production and altered BA pool composition. Reduced availability of FXR (CDCA) and TGR5 (LCA, DCA) agonists weakens tonic suppression of endothelial NF-κB—FXR via co-repressor recruitment and TGR5 via cAMP/PKA/*NLRP3* inhibition—leaving the endothelium more susceptible to pro-adhesive and pro-permeability signaling. Solid arrows denote experimentally established pathways; dashed arrows denote hypothesized downstream effects on vascular permeability. Abbreviations: 25HC, 25-hydroxycholesterol; BA, bile acid; cAMP, cyclic adenosine monophosphate; CDCA, chenodeoxycholic acid; CH25H, cholesterol 25-hydroxylase; CYP7A1, cholesterol 7α-hydroxylase; DCA, deoxycholic acid; FXR, farnesoid X receptor; HMGCR, 3-hydroxy-3-methylglutaryl-CoA reductase; HTNV, Hantaan virus; IFN, interferon; LCA, lithocholic acid; NF-κB, nuclear factor kappa B; *NLRP3*, NLR family pyrin domain containing 3; PKA, protein kinase A; TGR5, Takeda G protein-coupled receptor 5.
