# Supplementary material for: Bile acid signaling at the gut–vascular interface: a novel modulator of hantavirus endothelial barrier dysfunction
Source: Front Cell Infect Microbiol. 2026 Jul 22;16:1883162. doi: 10.3389/fcimb.2026.1883162 (PMC13437541; doi:10.3389/fcimb.2026.1883162)
Supplement: Supplementary file 1 [file Table1.pdf]

Supplementary Table S1. Established versus Hypothetical Components of the Proposed Gut Microbiota–Bile Acid–FXR/TGR5 Axis Hypothesis in Hantavirus Disease.

#	Statement	Status	Supporting Evidence
1	Hantavirus infection alters gut/lung microbiome in rodent reservoirs	Established	Brila 2023 (Ref 16), Xiong 2024 (Ref 17)
2	PUUV infects intestinal epithelium and disrupts ZO-1 tight junctions	Established	Witkowski 2017 (Ref 17)
3	GI symptoms are common in acute HFRS	Established	Clinical literature (Ref 18)
4	Monocyte-derived TNF-α/IL-6 drives endothelial barrier failure in HFRS	Established	Ref 6, 7
5	FXR is expressed in pulmonary endothelial cells	Established	Zhang 2012 (Ref 13)
6	FXR agonism suppresses NF-κB and protects against endothelial injury	Established	Ref 12, 13
7	TGR5 is expressed in monocytes/macrophages	Established	Ref 14
8	TGR5/cAMP/PKA inhibits NLRP3 inflammasome and suppresses TNF-α/IL-1β	Established	Ref 14, 17
9	HTNV induces CH25H→25HC→HMGCR→cholesterol biosynthesis↓	Established	Ref 29
10	Gut microbiome→BA pool composition changes occur in human HFRS	Hypothetical	No human HFRS microbiome data
11	BA pool perturbation→reduced FXR/TGR5 signaling in HFRS	Hypothetical	No BA metabolomics data in HFRS
12	Reduced FXR/TGR5→amplified endothelial barrier failure in HFRS	Hypothetical	Untested mechanistic prediction
13	FXR/TGR5 agonists could therapeutically attenuate HFRS severity	Hypothetical	No preclinical HFRS model data
14	GVI dysfunction amplifies TNF-α through BA pool shift	Hypothetical	Untested feedforward loop

Note: Ref numbers refer to the main manuscript reference list. "Established" indicates findings supported by published experimental or clinical evidence. "Hypothetical" indicates components that are mechanistically plausible but require direct experimental validation. Status assignments reflect the hantavirus disease context specifically; many individual mechanistic steps are well-established in other disease models.
